# Mobile Phones: The Next Step towards Healthcare Delivery in Rural India?

**DOI:** 10.1371/journal.pone.0104895

**Published:** 2014-08-18

**Authors:** Sherwin I. DeSouza, M. R. Rashmi, Agalya P. Vasanthi, Suchitha Maria Joseph, Rashmi Rodrigues

**Affiliations:** 1 St. John's National Academy of Health Sciences, Bangalore, India; 2 Department of Community Medicine, Saveetha Medical College, Thandalam, Chennai, Tamil Nadu, India; University Hospitals of Geneva, Switzerland

## Abstract

**Background:**

Given the ubiquity of mobile phones, their use to support healthcare in the Indian context is inevitable. It is however necessary to assess end-user perceptions regarding mobile health interventions especially in the rural Indian context prior to its use in healthcare. This would contextualize the use of mobile phone communication for health to 70% of the country's population that resides in rural India.

**Objectives:**

To explore the acceptability of delivering healthcare interventions through mobile phones among users in a village in rural Bangalore.

**Methods:**

This was an exploratory study of 488 mobile phone users, residing in a village, near Bangalore city, Karnataka, South India. A pretested, translated, interviewer-administered questionnaire was used to obtain data on mobile phone usage patterns and acceptability of the mobile phone, as a tool for health-related communication. The data is described using basic statistical measures.

**Results:**

The primary use of mobile phones was to make or receive phone calls (100%). Text messaging (SMS) was used by only 70 (14%) of the respondents. Most of the respondents, 484 (99%), were willing to receive health-related information on their mobile phones and did not consider receiving such information, an intrusion into their personal life. While receiving reminders for drug adherence was acceptable to most 479 (98%) of our respondents, 424 (89%) preferred voice calls alone to other forms of communication. Nearly all were willing to use their mobile phones to communicate with health personnel in emergencies and 367 (75%) were willing to consult a doctor via the phone in an acute illness. Factors such as sex, English literacy, employment status, and presence of chronic disease affected preferences regarding mode and content of communication.

**Conclusion:**

The mobile phone, as a tool for receiving health information and supporting healthcare through mHealth interventions was acceptable in the rural Indian context.

## Introduction

Wireless technologies now cover 96% of the global population and penetrate all walks of life. With 6.8 billion mobile-cellular subscriptions worldwide [1], the technology has successfully bridged gaps in communication and ignited economic growth and development globally [2]. It has also found a strong foothold within the healthcare sector in the emerging field of ‘mHealth’. ‘mHealth’ or ‘Mobile Health’ is healthcare supported by mobile technology, such as mobile phones, personal digital assistants and other wireless devices [3]. The emerging use of this technology in healthcare for treatment compliance, emergency management, mobile telemedicine, health promotion and community mobilization, are currently being researched or implemented globally [3].

### India and mobile communication technology

With 877 million wireless subscribers, India has the second largest wireless communication subscriber base in the world [4]. Wireless subscribers comprise 96% of telecom subscribers in India and contribute to an urban wireless tele-density fourfold that of rural India [4].

One of the reasons for the popularity of mobile phones in India is the low call tariff. At 1.6 USD/month, India has one of the lowest mobile call tariffs globally [5]. This makes mobile phone communication economical in the Indian context. Further, the average expenditure on mobile phones in rural households is an estimated five INR/month, while the same is 37 INR/month in urban poor households. More recent studies indicate that majority of the urban poor households in India spend approximately 3% of their monthly income on mobile communication [5]. Given the overwhelming popularity of mobile phone communication, at low cost, mHealth in the Indian context holds promise.

Research studies have explored the acceptability of mHealth interventions for supporting adherence to antiretroviral therapy in South India and for healthcare consultation in rural North India [6], [7]. The potential of mHealth is being harnessed by the Indian government in the ‘Mother and Child Tracking System’ (MCTS) within the ‘National Rural Health Mission’ (NRHM) [8], [9]. The MCTS gathers health information from antenatal and postnatal women in an attempt to ensure healthcare delivery to these women and to under-five children. Text messaging or Short Message Service (SMS) technology is also used to communicate with 3.2 million Indian central government employees under the Central Government Health Scheme (CGHS). Plans for its use in adolescent health, reproductive health and family planning, substance abuse and non-communicable disease prevention and treatment, are underway [10]. Given that the use of mobile phones, as a mode of communication in healthcare is inevitable, it is necessary to assess rural end-user perceptions and experiences with the technology. This would help contextualize healthcare delivery via mobile phones to 70% of the country's population residing in rural India.

We undertook this study to explore the acceptability of delivering healthcare interventions via mobile phones in a village in South India. Information obtained with this study could aid the development of appropriate user-friendly applications contextualized to the health needs of the population they target.

## Methods

This is an exploratory study done at a village, 52 kilometres from Bangalore, Karnataka State, South India between March 2009 and 2010. Karnataka state has a total population of 61 million. With 29,098 villages, the state has a rural population of 37,469,335 (61%). The Government provides primary healthcare to the rural population at no cost through a network of sub-centres, primary healthcare centres and community health centres. Additionally, healthcare can be availed at any of the several private facilities that may cater to the rural population for a fee. Healthcare at the village where the study was conducted, is available through a public primary healthcare facility and a private non-profit, faith-based secondary healthcare facility. This private healthcare facility supports research and medical internship training for medical interns from St. John's Medical College, Bangalore, South India, the academic institution that the researchers are affiliated with. The hospital also provides a base for some of the research work undertaken by the institution.

The village has an agrarian economy and is easily accessible by road via the national highway. It has a total population of 3,180 persons belonging to 608 households. Our survey covered all households in the village. Data was collected by door-to-door visits by trained members of the research team. A consenting participant above the age of 15 years, who owned a mobile phone, was enrolled in the study from each household visited. Those households found locked when visited, were excluded from the study.

An interviewer-administered questionnaire was developed. Some questions were adapted from the HIVIND study questionnaire that explored mobile phone usage in people living with HIV/AIDS at an infectious disease clinic in Bangalore, India [7]. The questionnaire was pretested in the local language i.e. Kannada and suitably modified based on feedback from the respondents to improve comprehension. The questionnaire assessed the respondent's demographic profile, mobile phone usage patterns and the acceptability of healthcare interventions delivered via mobile phones. The questionnaire included five domains (i) basic functionality of the mobile phone (ii) delivery and acceptability of health information via mobile phones (iii) use of mobile phones in the management of chronic illnesses (iv) use of mobile phones in the management of acute illnesses and (v) acceptability of usage of cell phones for health promotion.

### Data Analysis

Data was analysed using IBM-SPSS version 20. Frequencies, means, and standard deviation were used to describe the variables. Chi-square, Kruksal Wallis test and bivariate logistic regression models (standard LR) were used to study associations between demography and outcome variables, i.e. (i) preference for voice call to SMS reminders, (ii) more frequent versus less frequent medication reminders in chronic illnesses and (iii) preference for calling a doctor over the mobile phone in times of acute illnesses.

### Ethics statement

Ethical clearance for the study and its informed consent procedures was obtained from the Institutional Ethics Committee, St. John's Medical College, Bangalore, India, a private, non-profit, tertiary level, teaching, healthcare institution, to which the researchers were affiliated. Verbal consent was obtained from all potential participants or their guardians (for those <18 years of age) in the presence of a witness who endorsed the process. In addition, assent to participate in the study was obtained from participants below 18 years of age. Verbal consent was uniformly administered to all potential participants, some of who were illiterate, to ensure participation from those hesitant to sign the consent form but willing to participate in the study. This enabled representation of all demographic subgroups in the study.

## Results

Of the 608 households, 558 were available and willing to participate in the study, of these only 488 owned a mobile phone and were enrolled. The demographic details of the participants who owned a mobile phone are described in table 1. Those who owned a mobile phone had a larger median family than those who did not (Median family size 5; IQR 2 versus 4; IQR 2, p-value Kruksal Wallis test <0.004).

**Table 1:** Demographic characteristics of the study population **(n = 488)**.

	Total (%) (n = 488)	Female (%) (n = 360)	Male (%) (n = 128)
**Age**			
Median (Inter Quartile Range)	30 (25–40)	30 (25–37)	33 (25–45)
<20 yrs	30 (61)	28 (7)	2 (2)
20–40 yrs	328 (67)	254 (70)	74 (57)
>40 yrs	130 (27)	78 (22)	52 (4)
**Formally education** #			
No	29 (6)	21 (6)	8 (6)
Yes	360 (74)	339 (94)	120 (9)
**Literate in English**	46 (9)	26 (7)	20 (16)
**Currently employed**			
No	299 (61)	297 (83)	2 (2)
Yes	189 (39)	63 (17)	126 (98)
**Landline**	40 (8)	27 (7)	13 (10)
**Expenditure on mobiles** Median (IQR)	100 (IQR: 50–200)	100 (50–200)	100 (52–200)
**Socio economic status (n = 192)** $			
High	12 (6)	9 (7)	3 (4)
Middle	93 (48)	62 (50)	31 (46)
Low	87 (45)	53 (43)	34 (50)
**Family's with children**	84 (44)	50 (59)	34 (40)
**Chronic illness reported**			
No	448 (92)	336 (93)	112 (88)
Yes	40 (8)	24 (7)	16 (13)

# Formal education i.e. school and college education.

$ Socio economic status as defined by Parasuraman et al (1999).

### Basic functionality of mobile phones

All the 488 respondents routinely used their phones for making and receiving calls. On average, the respondents received four calls, while six outgoing calls were made in a day. Additionally, 70 (14%) used their mobile phones for text messaging, 56 (11%) for setting alarms, 337 (69%) for listening to music, 72 (15%) for playing games, 22 (4.5%) for photography and five (1%) for accessing the Internet. Those who were literate in English and 40 years of age or less were more likely to use the text messaging function than those who were not (unadjusted OR = 7.5, CI = 3.69, 15.14).

Of the 488 respondents who owned mobile phones, 484 (99%) were willing to make an appointment at the doctor's clinic via the mobile phone and 467 (96%) were willing to share their mobile number with their doctor. The 21 (4%) respondents who declined to share their phone number did so for fear of misuse of contact details or apprehension with regard to speaking with their doctor over the phone. Others preferred to meet the doctor in person.

### Mobile phones in health promotion

**Figure 1:**
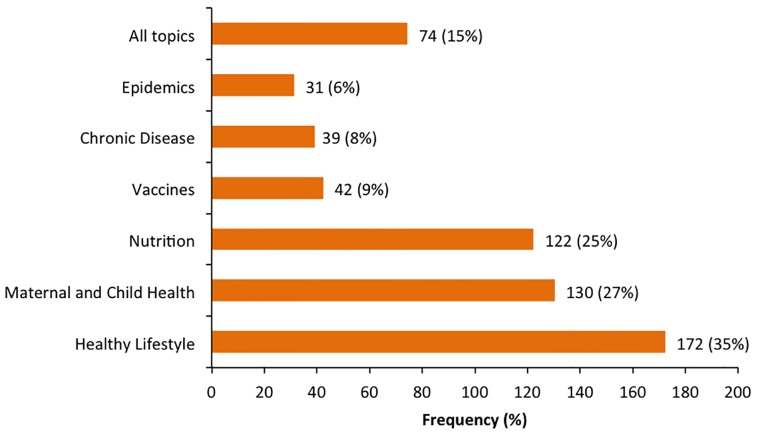
Type of health information requested over mobile phone (n = 488). (Uploaded as Figure 1 in TIFF format).

Of 488 respondents, 484 (99%) were open to receiving health information on mobile phones. Topics that participants preferred information on included healthy living, nutrition, maternal and child health, vaccination, self-care in chronic illnesses and information on infectious disease epidemics, (Figure 1).

Of the 484 respondents willing to receive health information via mobile phones, 218 (45%) preferred to receive the information daily, 223 (46%) weekly, and 42 (9%) monthly. A majority 371 (76%) preferred to receive vaccination reminders a day earlier to the date of vaccination, 73 (15%) on the vaccination day itself and 42 (9%) from a week to a month prior to the date of vaccination.

### Mobile phones in the management of chronic illnesses (table 2 and 3)

**Table 2:** Preference for daily or less frequent medication reminders compared to demographic characteristics **(n = 479)**.

	Prefer less frequent reminders (%) (n = 187)	Prefer more frequent reminders (%)(n = 292)	Unadjusted OR	Adjusted OR
**Age**	31.5±9.91	33.13±11.09	1.014 (0.997, 1.032)	1.014(0.995, 1.033)
**Sex**				
Female	151 (43)	202 (57)		
Male	36 (29)	90 (71)	1.868 (1.203, 2.902)	1.502 (0.780, 2.892)
**Formally educated** #				
No	8 (28)	21 (72)		
Yes	179 (40)	271 (60)	0.578 (0.250, 1.330)	0.589 (0.250, 1.391)
**Literate in English**				
No	176 (40)	259 (60)		
Yes	11 (25)	33 (75)	2.038 (1.003, 4.141)	2.264 (1.050, 4.886)
**Currently Employed**				
No	129 (44)	165 (56)		
Yes	58 (31)	127 (69)	1.712 (1.163, 2.250)	1.198 (0.670, 2.143)
**Chronic disease**				
No	168 (38)	274 (62)		
Yes	19 (51)	18 (49)	0.581 (0.296, 1.138)	0.459 (0.227, 0.930)

Logistic Regression Model p-value <0.001.

# Formal education indicates education that includes school.

**Table 3:** Preference for voice calls only and SMS with or without voice call reminders compared to demographic characteristics **(n = 480)**.

	Prefer voice calls only (%) (n = 424)	Prefer SMS or SMS + voice calls* (%) (n = 56)	Unadjusted OR	Adjusted OR
**Age**	33.19±10.85	27.48±7.64	0.937 (0.905, 0.970)	0.951 (0.917, 0.987)
**Sex**				
Female	314 (88)	40 (12)		
Male	110 (87)	16 (13)	1.142 (0.615, 2.120)	0.534 (0.230, 1.243)
**Formally educated** #				
No	26 (90)	3 (10)		
Yes	398 (88)	53 (12)	1.154 (0.337, 3.944)	0.575 (0.158, 2.091)
**Literate in English**				
No	395 (91)	37 (9)		
Yes	29 (60)	19 (40)	8.196 (4.131, 16.25)	4.579 (2.111, 9.933)
**Currently Employed**				
No	271 (92)	24 (8)		
Yes	153 (83)	32 (17)	2.361 (1.342, 4.156)	2.628 (1.209, 5.714)
**Presence of chronic disease**				
No	396 (89)	48 (11)		
Yes	28 (78)	8 (22)	2.270 (0.981, 5.248)	1.661 (0.642, 4.296)

Logistic Regression Model p-value <0.001.

# Formal education indicates education that includes school.

*This category includes those who preferred the SMS alone or both SMS and voice calls.

AIC: 312.7853, BIC: 342.002.

For the management of chronic illness, 479 (98%) respondents preferred to receive medication adherence reminders via mobile phones. Those who refused reported that they remembered to take their medication without reminders. From among those who preferred reminders, 424 (89%) preferred only voice calls, 45 (9%) preferred text messages and 11 (2%) had no specific preference. Most respondents preferred voice calls (419; 86%) and SMSs (319; 65%) in the local language i.e. Kannada. With every year increase in age, the preference for SMSs was less likely in comparison to voice calls (adjusted OR = 0.951, CI = 0.917, 0.987). Those who were literate in English (adjusted OR = 4.579, CI = 2.111, 9.933) and those currently employed (OR = 2.628, CI = 1.209, 5.714) were more likely to prefer SMSs alone or SMS and voice calls in comparison to those who were not literate in English and those unemployed.

Medication reminders were preferred as often as the medication was to be taken by 163 (34%), daily by 129 (27%), biweekly by 22 (5%) and weekly by 165 (34%). Respondents who were literate in English were twice more likely to prefer more frequent reminders (adjusted OR = 2.264, CI = 1.050, 4.886), while those who suffered from chronic illnesses were less likely to prefer more frequent reminders (adjusted OR = 0.459, CI = 0.227, 0.930). We also found that men were almost twice more likely to prefer more frequent medication reminders in comparison to women (unadjusted OR = 1.868, CI = 1.203, 2.902), as were respondents who were currently employed (unadjusted OR = 1.712, CI = 1.163, 2.250).

### Mobile phones in the management of acute illness

Of the 488 respondents, 367 (75%) were willing to call their doctor using their mobile phones for the management of an acute illness and 487 (99.7%) would call their doctor with their mobile phones in a medical emergency. Respondents with a formal education were 6 times more likely to call their doctor over the mobile phone in an acute illness (adjusted OR = 6.866, CI = 3.080, 15.30) as opposed to those literate in English who did not prefer calling the doctor (adjusted OR = 0.114, CI = 0.055, 0.236), (table 4). Further, men were less likely to prefer calling their doctor in the management of an acute illness in comparison to women (unadjusted OR = 0.606, CI = 0.388, 0.948).

**Table 4:** Preference for calling the doctor over the mobile phone for acute illnesses compared to demographic characteristics **(n = 488)**.

	Did not prefer calling the doctor (%) (n = 121)	Preferred calling the doctor (%) (n = 367)	Unadjusted OR	Adjusted OR
**Age**	31.89±10.68	32.72±10.64	1.007 (0.988, 1.027)	1.00 (0.979, 1.025)
**Sex**				
Female	80 (22)	280 (78)		
Male	41 (32)	87 (68)	0.606 (0.388, 0.948)	1.073 (0.527, 2.183)
**Literate in English**				
No	89 (20)	353 (80)		
Yes	32 (70)	14 (30)	0.110 (0.056, 0.218)	0.114 (0.055, 0.236)
**Formally educated** #				
No	17 (59)	12 (41)		
Yes	104 (23)	355 (77)	4.835 (2.238, 10.450)	6.866 (3.080, 15.30)
**Currently Employed**				
No	57 (19)	242 (81)		
Yes	64 (34)	125 (66)	0.460 (0.303, 0.698)	0.593 (0.307, 1.143)
**Chronic disease**				
No	108 (24)	342 (76)		
Yes	13 (34)	25 (66)	0.607 (0.300, 1.228)	0.822 (0.366, 1.844)

Logistic Regression Model p-value <0.001.

# Formal education indicates education that includes school.

Respondents who did not prefer using their mobile phones in an emergency, did not do so, either due to the proximity of the hospital or because they preferred to consult a doctor in person.

### Challenges to the use of mobile phones in healthcare

From among 488 respondents, 475 (97%) felt that receiving health information via mobile phones was not an intrusion into their lives. On the contrary, 345 (70%) felt that calling their doctor over the phone would disturb the doctor at work.

Prejudice that the mobile phone was a bad influence on the youth and concerns about the health hazards of mobile phone usage, expressed by 2 of the respondents, were potential barriers to their use in healthcare.

## Discussion

Rapid advances in mHealth call for the development of end-user friendly mobile phone applications that may be used for healthcare delivery. These applications should be simple, minimally intrusive and ensure confidentiality of personal information. It is equally important to contextualize every planned intervention to the population for which it is intended. With the Indian government's new impetus to use mobile phone technology in healthcare, mHealth services are expected to have a vast rural outreach. Given the immense potential for mHealth in India, we chose to explore experiences and perceptions of rural Indian mobile phone users towards using mobile phone technology in healthcare.

### Mobile phone-based reminders

A majority of respondents expressed interest in receiving medication adherence reminders for chronic illnesses. Forgetfulness, a barrier to medication adherence [11], can be minimised with the use of reminders. Medication reminders can be sent via mobile phones. Such reminders have been found effective in improving medication adherence, in chronic non-communicable disease and communicable diseases like tuberculosis and HIV infection [12]–[18].

Further, our respondents also expressed an interest in receiving appointment reminders and vaccination reminders. Reviews indicate appointment reminders via SMS improve attendance in primary care clinics, chronic disease follow up, family planning clinics and ophthalmology clinics [19], [20]. A study from rural Haryana, reported using mobile phones to obtain appointments for outpatient visits [21]. The probable reduction in clinic waiting time is assumed to have made the concept more appealing to our respondents.

### Preferred type of communication: *SMS versus Voice calls*

The second global survey on eHealth reported that SMS reminders were preferred to voice calls, globally [3]. However, most of our respondents preferred voice calls to text messages. Employed or English-literate respondents were more likely to prefer SMS communication. The employed respondents, probably due to lesser personal time and privacy at work, preferred reading an SMS at their own convenience as opposed to answering a phone call.

A study from Mumbai, India reported that only a few women at an urban antenatal clinic reported using the SMS facility. Their reasons included low literacy levels and even among the literates, difficulty in articulating text messages [22]. Similarly, technical difficulties in responding to both IVR calls and SMS reminders have been reported from South Africa [23]. Voice calls may be more useful in a population with minimal expertise in mobile phone operation or for health conditions that require more direct interaction e.g. suicide hotlines, HIV helplines, contraceptive hotlines [24]–[26]. The preference for voice calls among older respondents indicates a probable discomfort in operating mobile phones to access an SMS or a difficulty with reading SMSs. It is likely that some of our respondents also found SMS technology cumbersome to use. While SMS technology may have greater appeal among the English literate, maintaining communication in the local language could make them more accessible to those literate only in the local language. It is also worth noting that though the language maybe different, the script of communication is frequently English. This may continue to be a barrier to the use of SMS technology. Studies on HIV-infected populations in our setting (South India) have reported similar preferences for voice calls and an association with English language literacy and the preference for voice calls [7], [18], [27].

Despite SMS not being preferred in our setting, it has been found acceptable and effective elsewhere. Hospital attendance rates of patients receiving SMS reminders were comparable with those receiving voice call reminders, with lower cost per attendance [28]. Vaccination reminders (text messages) were found more acceptable than phone calls and letters among Latino mothers in New York City [29] and improved influenza vaccination rates in children and adolescents in low-income urban populations in USA [30]. In populations with low literacy, the possibility of using pictorial SMS reminders that use standard symbols e.g. ASCII art, could be explored [31]. It is therefore our recommendation that in the Indian context, voice calls would be the best form of communication. Failing this option, pictorial text messages using universally understood symbols maybe used in an effort to abstain from using the English script.

### Frequency of reminders

While language and format are obvious issues, increased frequency of reminders is a significant cause of intervention-fatigue and therefore needs attention [32], [33]. Respondents literate in English were twice more likely to prefer more frequent reminders. This may be explained by the fact that they were also more likely to be employed (p<0.001) or involved in completing their education and therefore likely to forget their medication and would benefit from frequent reminding. By similar reasoning, men and those employed were also more likely to prefer more frequent reminding in comparison to women and those unemployed. Respondents with chronic disease may have already become accustomed to maintaining a medication schedule without any assistance, therefore requiring less frequent reminders.

### Use of mobile phones in behaviour change communication

Mobile phones have been used in creating health awareness and bringing about behavioural change [12], [34]. SMS has been successfully used to improve physical activity and reduce the number of servings of red meat in middle-aged men in Australia [35] and receiving information on lifestyle modification lowered the incidence of diabetes in men (30–35 years of age) with impaired glucose tolerance in India [36]. A majority of our respondents also consider the mobile phones an acceptable tool for health education. Mobile phone based communication therefore provides an opportunity to promote a healthy lifestyle while satiating the desire for health information in our study population [37].

Our respondents also expressed interest in information on maternal health and child. Studies exploring an SMS intervention to promote healthy behaviour during pregnancy showed improved birth-preparedness and healthy attitudes to alcohol consumption in a pilot study in Virginia, USA [38]. SMSs were also considered acceptable for sexual health promotion in different settings [39], [40]. Similar communication to educate women about antenatal care, maternal nutrition, necessity of iron and folic acid (IFA) tablet consumption and newborn care could be explored in the rural Indian context. The possibility of improving adherence of IFA tablets and maintaining obstetric appointments through reminders should also be considered.

Health information could be made available passively through text and automated voice calls or a system where people call a phone number, select the type of information they want by keying in codes for a particular topic and listen to pre-recorded information regarding the same. Such automated systems may however be difficult to operate [23]. Live helplines could provide real-time assistance in addition to a personal touch. Reports of switching from automated HIV helplines to those manned by personnel are available from Bangalore, India [41]. However, establishing and maintaining them is resource-intensive, in comparison to automated communication systems [25].

### Use of mobile phones in acute care and epidemics

A study from Washington, D.C. reported participants' willingness to send photographs of their wounds to physicians for diagnosis and recommendations [42]. Majority of our respondents were willing to communicate with healthcare providers via mobile phones in an emergency. However, their limited use of phone camera (4.5%) and phone-based Internet (1%) minimises the possibility of using photographs, Internet and MMS. A study from Nakuru, Kenya demonstrated that mobile phones were useful in facilitating communication and decision-making in reproductive health [40]. This may be due to quicker communication and easier access to information in an emergency. Such use of mobile phones could especially benefit rural India, where frequently, patients must travel long distances to meet a doctor, not only for their most basic health requirements but also in emergencies. The possibility of using emergency helplines that either provide verbal basic or professional assistance could also be explored in this setting. However for the population in our study, accessing healthcare in an emergency may not pose a significant issue given the presence of a primary and a secondary level healthcare facility in the village.

In our study, those with formal education were more likely to contact their doctor via mobile phone, for acute and emergency care. It is possible that this group had better health literacy [43] than the others because of their education. They were likely to understand the importance of health personnel and urgent intervention in the management of emergencies. However, those literate in English were less likely to contact their doctor in an emergency. The reasons for this association need to be determined.

A study in China used mobile phone technology in a disaster management setting for detecting and controlling disease outbreaks [44]. Data gathering via mobile phones, was done effectively in malaria epidemics in Sub-Saharan Africa [45]. A study from Ivory Coast, Africa reported the use of mobile phone-based one-to-one communication to create awareness and control epidemics [46]. Our respondents were also open to such usage of mobile phones in the setting of infectious epidemics. However, care must be taken to ensure communication is informative while not creating panic regarding the epidemic.

### Privacy and related concerns

While most participants were open to using mobile phones for health-related issues, those who were not, generally preferred face-to-face interactions with their healthcare provider. A substantial proportion felt they would be invading their doctors' privacy by calling him or her on the mobile phone. Some of our respondents expressed concern with sharing their phone number with the doctors' clinic. However, we did not assess specific privacy and confidentiality concerns. Studies report minimal privacy concerns for communicating laboratory results to HIV patients and wound images for acute healthcare [42]. Universally acceptable guidelines addressing confidentiality, privacy and ethical concerns applicable to mHealth and adaptable to local contexts need to be developed.

### Women, health and mobile phones

A large proportion of the respondents in our study were women with access to mobile phones. In the Indian context, women are often responsible for the health and hygiene in their family. They are primarily involved in cooking, cleaning and caring for children and elderly in their households [47]. It is therefore not surprising that nutrition and maternal and child health were popular among information requested. The existing experience with caregiving within families may have resulted in women preferring less frequent adherence reminders in our study. Women were also less likely to be employed making their schedules more flexible and conducive to ensuring better medication adherence in comparison to men [32]. Our finding that women were more likely to communicate directly with their doctor in the management of an acute illness may indicate that they had lesser knowledge about health issues and needed assistance. It is also noteworthy that they were able to take cognizance of a situation where they were out of their depth, and were willing to assume responsibility in procuring the necessary expertise required.

Thus, while reduced autonomy among majority of Indian women is likely to adversely affect health-seeking behaviour; our findings indicate that mobile phones may serve to enable women to actively participate not only in their own healthcare and but also that of their families [47]. Our study was able to capture the opinions of women, a group largely underrepresented in research, since the interviews were conducted primarily during working hours. Given that the women in our study have access to mobile phones, both the women and the healthcare system have an opportunity to communicate with each other, which can be exploited to improve the population's health.

### Methodological issues

Our study assessed the usage and acceptability of mobile phones in healthcare in a rural setting approximately 52 kilometres from Bangalore city. It is possible that participant responses may not be entirely representative of other rural settings in India, given the proximity of our study setting to Bangalore city.

Healthcare personnel, who routinely administer services to the concerned community, were responsible for data collection. Therefore the possibility of social desirability bias or acquiescence must be considered. Further, as most of the interviews were conducted during the day, the opinions of those who are regularly away during these hours may not be adequately reflected. This also explains the large proportion of women in our study. Keeping in mind that we tried to evaluate the acceptability of a new form of communication; we must consider, having included the opinions of more forthcoming respondents, as a possible bias. No specific data on the type of mobile phones or privacy concerns with mobile healthcare communication was obtained in our study. Though the acceptability of reminders for supporting medication adherence was assessed, we did not assess their acceptability for supporting adherence to prescribed diet and exercise. Further, as missing data on socio-economic status reduced the size of the dataset this variable was excluded from the analysis.

## Conclusion

Our study sought an answer to the question of whether mobile phones would be an acceptable next step towards improving healthcare delivery in India. Our findings have generally corroborated the acceptability of mHealth interventions and may even direct future endeavours in this area.

mHealth interventions such as reminders and information disseminating applications via mobile phone were acceptable in our study. The voice call was the preferred mode of communication in our setting, and needs to be considered in light of the popularity of SMSs globally. Attention to factors such as English literacy, education, employment status, and sex of the end user would only serve to improve the efficacy of mhealth. Healthcare communication directed at women via mobile phones, could empower them with the necessary knowledge to promote not only their own health but also the health of their families. While these findings are encouraging to further the development and deployment of mHealth interventions in rural India, the interventions designed should be acceptable to the targeted population and minimally intrusive while ensuring the privacy of the end user.

## Supporting Information

Annexure S1 **Questionnaire - Mobile phones in health care in rural India.** This is the questionnaire that was developed for the purpose of the study.
(DOCX)
