# Supplementary material for: Mobile Phones: The Next Step towards Healthcare Delivery in Rural India?
Source: PLoS One. 2014 Aug 18;9(8):e104895. doi: 10.1371/journal.pone.0104895 (PMC4136858; doi:10.1371/journal.pone.0104895)
Supplement: Annexure S1 — Questionnaire - Mobile phones in health care in rural India. This is the questionnaire that was developed for the purpose of the study. (DOCX) [file pone.0104895.s001.docx]

**SURVEY ON USAGE OF MOBILE PHONES IN FOR HEALTHCARE IN RURAL INDIA**

**Date of Interview: Interviewer:**

**Family Details:**

|  | **Number of family members:**  **Number of adults:**  Male:  Female:  **Number of children:**  Male  Female: | **Number of mobile phones owned:**  In the entire household:  By the males: ___  By the females: ___  **Do you own a landline phone connection:** Yes/No    **Amount of money spent on the phones per month (in INR):**  Landline: ________  Mobile phone: ________ |
| --- | --- | --- |
|  |  | **SLI (Standard of Living Index):** |

1. **BIOGRAPHY:**

| 1 | Name: | 7 | Education level:    a) No formal b) Primary school  c) Middle school d) High school  e) Pre-University f) Degree  g) Post -graduate |
| --- | --- | --- | --- |
| 2 | Age: | 8 | Occupation:  a) Professional b) Semi-professional c) Skilled d) semi-skilled e) Unskilled |
| 3 | Gender: Male/ Female | 9 | Address & telephone number: |
| 4 | Marital Status:  a) Married living with spouse  b) Married but separated  c) Divorced  d) Not married  e) Living alone | 10 | Diagnosis with illness, (if any): |
| 5 | Language (mother tongue): | 11 | Are you currently receiving any treatment for the above-mentioned illness:  Yes/No. |
| 6 | Languages known:  (To speak only)___________________________  (To read and write)________________________ | 12 | If receiving treatment, details of medication: |

1. **KNOWLEDGE ON MOBILE PHONES:**

| 13. | Do you routinely use mobile phones?  Yes / No  If ‘yes’ go to question 15 directly | 19. | How often do you:   1. Call others: ________per day or per week 2. Receive calls: _______per day or per week |
| --- | --- | --- | --- |
| 14. | What prevents you from using your mobile phone?  (Multiple response possible)   1. Lack of money 2. Lack of network 3. Have no purpose for which to use the mobile phone 4. Unable to use the mobile phone 5. Other reason. Please specify________ | 20 | Do you use the mobile phone to SMS?  Yes/No  If No skip to 34 |
| 15. | Do you own a mobile phone?  Yes/No.  If ‘No’ skip to 34. | 21 | How often do you,  a. Send an SMS: ______per day or per week  b. Receive an SMS: ______per day or per week |
| 16. | Is this phone usually in your possession?  Yes/ No.  If ‘no’ shared by_______. | 22 | Do you use the alarm function on your mobile phone?  Yes/ No.  If no skip to 24 |
| 17 | Since when have you used a mobile phone?  _________ Years.  (Fill in years. Eg: 0.5, 1, 1.5 etc.) | 23 | For what do you use the mobile phone alarm function? (Multiple answers possible)   1. As a wake up call 2. To remind me of errands 3. As a reminder for medication 4. Other________ |
| 18 | Do you make or receive calls via your mobile phone?  Yes/ No  If no, skip to 20 | 24 | For what else do you use the mobile phone? (Multiple answers possible)   1. For listening to radio/music 2. For playing games 3. To use the camera 4. To access the internet 5. Others uses________ |
|  |  | 25. | Do you have landline facility?  Yes/ No |

**Usage Of Mobile Phones In Health Promotion:**

| 26 | Would you like to provide your mobile number to doctor so that he or she may contact you?  Yes/ No | 30 | How often would you like to receive this information?   1. Daily 2. Once a week 3. Once a month |
| --- | --- | --- | --- |
| 27. | If you responded ‘no’ to the previous question, please explain your reasons? | 31 | Would you like to receive alerts about vaccination days, health camps in the area in which you live?  Yes/ No. |
| 28 | Would you like to receive health advice on your mobile phone?  Yes/ No | 32 | Would you like to receive reminders for vaccination for your child?  Yes/ No. |
| 29 | If yes, what topics would you like receiving information on?   1. Nutrition 2. Healthy life style 3. Pregnant woman’s health 4. Mother and Child health 5. Vaccinations 6. Self care    1. E.g.: self care in chronic diseases like hypertension and diabetes    2. Self care during pregnancy etc 7. Information on epidemics and precautions    1. Ex: Swine flu, Chikungunya 8. Others __________. | 33 | If yes, when would you like to receive the reminder?   1. One day before the date of vaccination 2. On the day of vaccination 3. One week/ month earlier to vaccination |

1. **Usage Of Mobile Phones In Management Of Chronic Illness:**

| 34 | Consider a patient with a chronic illness such as diabetes mellitus or hypertension, currently receiving medications. Do you think it would be helpful to have automatic reminders on the mobile phone to help them remind to take medicines?  Yes/ No.  If ‘No’ skip to 40 | 38 | If we were to provide patients with automatic reminders for their medication, how often would you like these reminders? (Choose only one.)   1. As often as the medicine needs to be taken 2. Daily 3. Once a week 4. Twice a week |
| --- | --- | --- | --- |
| 35 | If we were to provide you with automatic reminders for taking your medications, what format would you like these reminders to be? (Choose only one response)   1. Phone call 2. SMS 3. No preference | 39 | Why do you think this frequency of reminding would be convenient for the patient?  Skip to 21. |
| 36 | If the reminder were in SMS form, in which language would you like the SMS?   1. English 2. Kannada (local language) 3. Other_____ | 40 | Why would you think these medication reminders are not useful? Write reasons. |
| 37 | If the reminder were in telephone call form, in which language would you like the telephone call?   1. English 2. Kannada 3. Other | 41 | If we were to develop a mobile phone based application for persons living with chronic illnesses, what would you like to see this application do?   1. Enable communication with counsellor/ health worker 2. Provide Information on medicines 3. Provide further information about illnesses 4. Others uses |

1. **Usage Of Mobile Phones In The Management Of Acute Illness:**

| 42 | If you possessed a mobile phone, would you use it to communicate with your doctor or health worker?   1. Yes, definitely. 2. Yes, sometimes. 3. Not sure. 4. Very rarely. 5. Probably not. |  | Do you feel it would be beneficial to use mobile phones during emergencies like accidents?  Yes/ No.  If ‘No’ why? |
| --- | --- | --- | --- |
| 43 | Would you prefer to call a doctor over a mobile phone, for care of an acute illness?  Yes/ No.  If no ask why? | 46 | Do you feel that the use of mobile phones for medical care during emergencies could be beneficial?  Yes/ No.  If ‘No’ why? |
| 44. | Would you like to be able to make an appointment with your doctor over the phone?  Yes/ No  If ‘No’ why? |  |  |

1. **Mobile Phone Usage: Opinions**

| 47 | Do you think that usage of mobile phones to supplement health practices in the above-mentioned ways will intrude into a peoples’ lives?  Yes/ No | 49 | Do you think calling the doctor over the mobile phone will be troublesome to him/her?  Yes/ No |
| --- | --- | --- | --- |
| 48 | If yes, why? | 50 | If yes, why do you think so? |

**Comments:** __________________________________________________________
____________________________________________________________________
____________________________________________________________________

____________________________________________________________________
